# Mechanism of artemisinin resistance for malaria PfATP6 L263 mutations and discovering potential antimalarials: An integrated computational approach

**DOI:** 10.1038/srep30106

**Published:** 2016-07-29

**Authors:** Nagasundaram N., George Priya Doss C., Chiranjib Chakraborty, Karthick V., Thirumal Kumar D., Balaji V., Siva R., Aiping Lu, Zhang Ge, Hailong Zhu

**Affiliations:** 1 School of Chinese Medicine, Hong Kong Baptist University, Kowloon Tong, Hong Kong; 2 School of Biosciences and Technology, VIT University, Vellore 632014, Tamil Nadu 632014, India; 3 Department of Bioinformatics, School of Computer and Information Sciences, Galgotias University, Greater Noida, Uttar Pradesh, India; 4 Department of Clinical Microbiology, Christian Medical College, Vellore 632014, Tamil Nadu, India

## Abstract

Artemisinin resistance in *Plasmodium falciparum* threatens global efforts in the elimination or eradication of malaria. Several studies have associated mutations in the *PfATP6* gene in conjunction with artemisinin resistance, but the underlying molecular mechanism of the resistance remains unexplored. Associated mutations act as a biomarker to measure the artemisinin efficacy. In the proposed work, we have analyzed the binding affinity and efficacy between PfATP6 and artemisinin in the presence of L263D, L263E and L263K mutations. Furthermore, we performed virtual screening to identify potential compounds to inhibit the PfATP6 mutant proteins. In this study, we observed that artemisinin binding affinity with PfATP6 gets affected by L263D, L263E and L263K mutations. This *in silico* elucidation of artemisinin resistance enhanced the identification of novel compounds (CID: 10595058 and 10625452) which showed good binding affinity and efficacy with L263D, L263E and L263K mutant proteins in molecular docking and molecular dynamics simulations studies. Owing to the high propensity of the parasite to drug resistance the need for new antimalarial drugs will persist until the malarial parasites are eventually eradicated. The two compounds identified in this study can be tested in *in vitro* and *in vivo* experiments as possible candidates for the designing of new potential antimalarial drugs.

The medicinal use of artemisinin (qinghaosu) from sweet wormwood (*Artemisia annua*) was explored in 1970 by Chinese scientist; since then it serves as a primary chemotherapy agent in antimalarial treatment. The number of malaria patients curing with artemisinin combined therapies has been increased exponentially^1^ but the accurate molecular mechanism of action is still controversial^2^. Artemisinin is a sesquiterpene lactone endoperoxide containing a structural feature called peroxide bridge and assumed to be crucial for the mode of action^1^. Initial research suggests that the mechanism of action of artemisinin is by heme-dependent activation of an endoperoxide bridge occurring within the parasite’s food vacuole^3^,^4^. However, localization of artemisinin to the parasite and not food vacuole membranes^5^ and killing of tiny rings lacking haemozoin argue against the food vacuole being a major site of drug action^6^. Some other studies proposed a different mode of action of artemisinin, which is based on compound structural resemblance and the sesquiterpene moieties of thapsigargin. Thapsigargin is a plant product from *Thapsia garganica* shows structural similarities to artemisinin. Thapsigargin is considered as a highly selective inhibitor of Sarco/endoplasmic reticulum Ca^2+^-ATPase (SERCA). Based on this it was hypothesized that artemisinin may act in a similar way, but selectively to inhibit the SERCA of the malarial parasite. PfATP6 is the only SERCA-type Ca^2+^-ATPase enzyme present in the malarial parasite and it is considered to be the suitable molecular target for artemisinin^5^. An *in vitro* examination also suggests that artemisinin inhibit the SERCA-type Ca^2+^-ATPase orthologue (PfATP6) of *P. falciparum* in Xenopus oocytes^7^.

The successful progress of the elimination of malaria with artemisinin combination therapy is now hindered by the spreading antimalarial drug resistance. Artemisinin resistance is evident from slow parasite clearance^8^,^9^ and which affects the susceptibility of ring stage parasites^10^,^11^,^12^,^13^,^14^. In western Cambodia first artemisinin resistance was reported^8^,^9^ followed by resistance rates for artemisinin based combination therapies were exponentially increasing^15^. Artemisinin resistance is also declared in mainland Southeast Asia; here it emerges independently and started to spread rapidly^16^,^17^,^18^,^19^. Amino acid mutation at position 263 of PfATP6 enzyme tremendously affects the sensitivity of the enzyme to artemisinin and is located in the artemisinin binding pocket^20^. *In vitro* studies in *X. laevis* oocytes elucidated that the substitution of a single amino acid residue L263A or L263S resulted in an approximately three-fold increase or decrease in sensitivity to artemisinin. Particularly the substitution of L263E leads to complete abolishment of interaction by artemisinin^20^. However, this observation was not extended to *P. falciparum*, where introduction of the L263E mutation through transgenics resulted in borderline non-significant changes in the 50% inhibitory concentrations (IC50s) for artemisinin and its derivatives^21^. Recent report from Uhlemann *et al*. in 2012^20^ stated the reconfirmation of their previous conclusion that single amino acid mutations in PfATP6 can abolish sensitivity to artemisinin, as shown by the results obtained with PfATP6 mutants L263D, L263E and L263K. These observations hypothesised that mutations in the particular residue L263 residing in drug binding pocket might affect the affinity of the drug and consecutively cause decreased susceptibility to artemisinin.

In the proposed study, we carried out molecular modeling of malaria PfATP6 protein and molecular docking between wild type PfATP6-Artemisinin and mutant type (L263D, L263E and L263K)-Artemisinin to elucidate the detailed mechanism of binding action. Further structure-based virtual screening was performed to identify novel lead molecules to inhibit the mutant PfATP6 proteins. To ensure the stability of protein-ligand complexes 50 ns united atom molecular dynamics simulation was performed thrice for the wild type and mutant type PfATP6-Artemisinin and mutant PfATP6-Virtually screened compounds.

## Results

### Homology modeling of malarial PfATP6 protein

**Figure 1:**
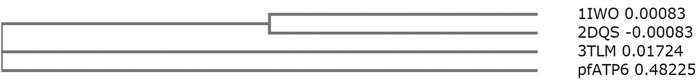
Phylogenetic tree. Phylogenetic tree shows the evolutionary relationship between malarial PfATP6 protein and template (3TLM, 1IWO, and 2DQS) protein sequences.

**Figure 2:**
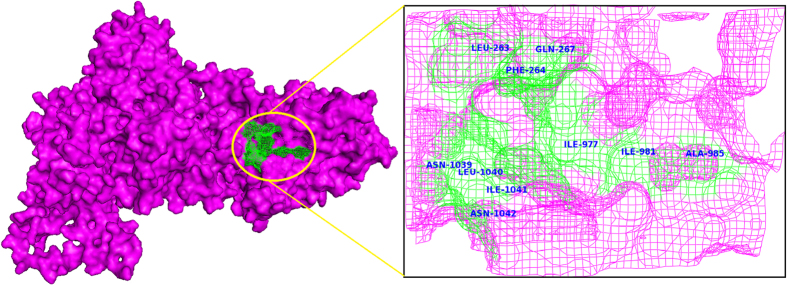
3D structure of homology modeled malarial PfATP6 protein highlighted with artemisinin binding residues.

**Figure 3:**
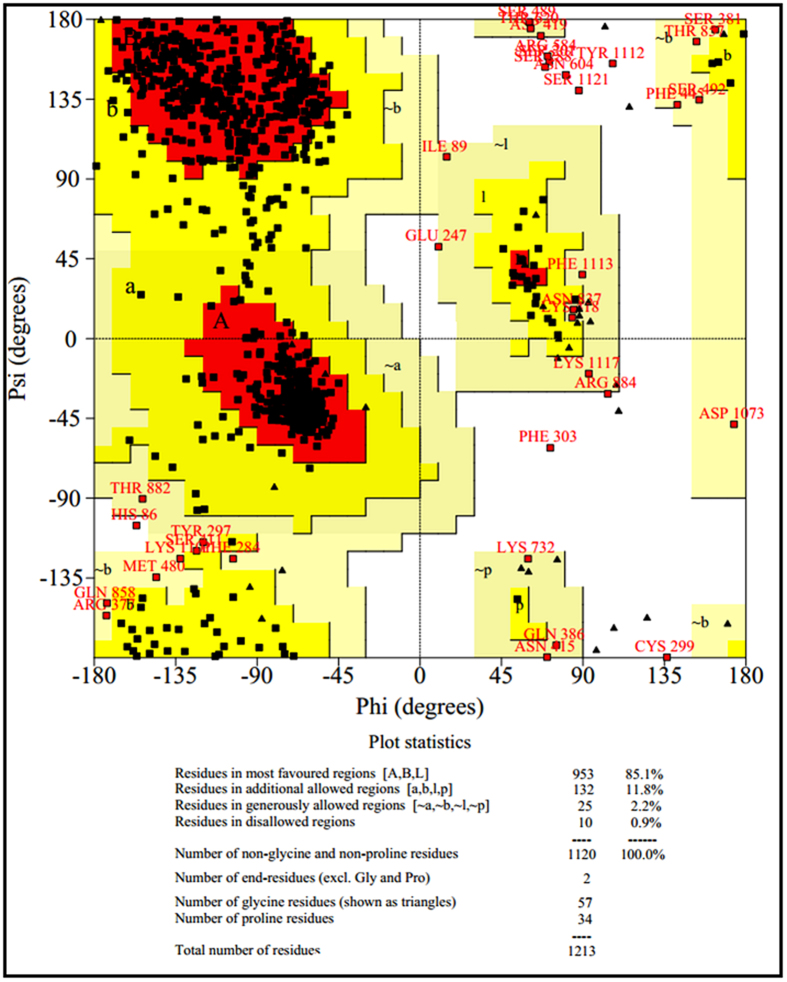
Ramachandran plot of a protein structure derived from homology modeling. It provides an overview of allowed and disallowed regions of torsion angle values, serving as an important indicator of the quality of protein three dimensional structures.

The protein sequence of PfATP6 is compossed of 1228 amino acids residues retrieved from the UniProt^22^ database. In BLAST sequence similarity analysis PfATP6 of *Plasmodium falciparum* obtained highest sequence homology (45% identity) with Endoplasmic Reticulum Ca^2+^-Atpase (SERCA) from the bovine muscle of Bos taurus. The tertiary structure of PfATP6 can be modelled by using the crystal structure of the SERCA homologue (PDB: 3TLM, resolution 2.95A°)^23^. Modelling of PfATP6 enzyme based on templates (1IWO and 2DQS) and docking of artemisinin derivative on to it was reported earlier by Jung *et al*. and Naik *et al*.^24^,^25^. They observed good correlation between the computational binding affinity results and *in vitro* antimalarial sensitivity. Both 1IWO and 2DQS templates are from the same organism Oryctolagus cunniculus bound with thapsigargin but having closed conformation of calcium pump. In order to select the best template for 3D model construction phylogenetic tree analysis was performed between malarial PfATP6, 3TLM, 1IWO and 2DQS protein sequences. From the phylogenetic analysis result it is observed that malarial PfATP6 protein sequence and SERCA protein sequence from the bovine muscle of Bos taurus are evolutionarily more close (Fig. 1). Moreover, the binding affinity achieved by several docking and scoring methods could not differentiate between inhibitor and non-inhibitor in closed model of PfATP6. In this study, our ultimate goal is to seek a plausible mechanism action of artemisinin binding with wild and L263 mutant PfATP6 enzyme. Hence we studied from the model which is built using open conformation template (3TLM) of the enzyme to see the dynamic effect of protein-ligand complex along with the solvent. The sequences of PfATP6 and the bovine muscle of Bos taurus SERCA were aligned using ClustalW 2.0.1^26^. The 3D structure of PfATP6 protein (Fig. 2) was constructed by using Modeller 9.15^27^,^28^. The quality of the refined PfATP6 model was assessed by PRO-CHECK^29^. The distribution of the Psi/Phi torsion angles of the best model is represented by a Ramachandran plot (Fig. 3), which shows 85.1% of residues are in most favored regions, 11.8% in additional allowed regions, 2.2% in generously allowed regions and 0.9% in disallowed regions. The calculated Ramachandran Z-score is 0.168, meaning that there is a good agreement between the PfATP6 model and the SERCA template.

### Docking analysis of artemisinin with wild and mutant (L263D, L263E and L263K) malarial PfATP6 proteins

**Figure 4:**
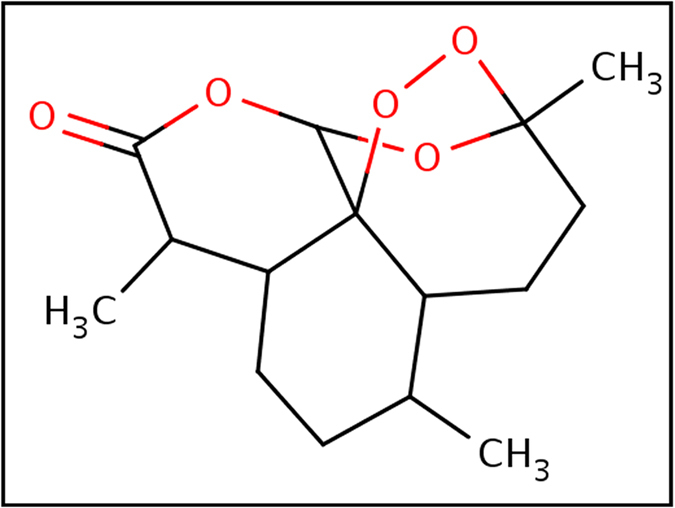
2D image of drug artemisinin.

**Figure 5:**
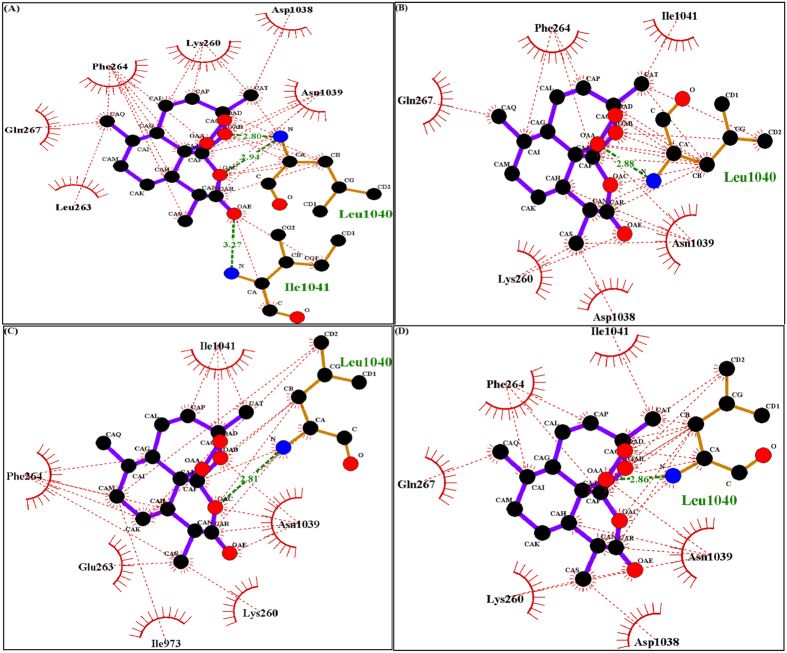
Ligplot analysis of PfATP6 wild type-artemisinin and mutant types -artemisinin complexes, green lines indicates the hydrogen bonds and red dotted lines indicates the hydrophobic interactions. (**A**) Figure showing the wild type PfATP6 protein interacting residues with drug artemisinin. (**B**) Ligplot showing the interaction between mutant model L263D and artemisinin. (**C**) Ligplot showing the interaction between mutant typeL263E and artemisinin. (**D**) Ligplot showing the interaction between mutant typeL263K and artemisinin.

Artemisinin (Fig. 4) has been suggested to inhibit the *in vitro* activity of the malarial PfATP6 protein^30^,^7^. Several studies have been attempted to find the binding mechanism of artemisinin with malarial PfATP6 protein^23^,^24^. Additionally protein structure based drug design approaches have followed to optimise inhibitor binding specificity^25^,^31^. In this study, we explored the appropriate binding conformation and affinity of artemisinin with wild and mutant PfATP6 proteins using a computational docking program Auto Dock Vina^32^. The PfATP6 protein’s ligand binding residues were identified with thapsigargin as the reference ligand. The ligand binding site is close to the centre of the four transmembrane helices. Even minor conformational change in these helices could affect the Ca^2+^ binding sites and there by change the movement of ions to the luminal spaces or cytosol^33^,^34^,^35^. The active site residues of PfATP6 consists of LEU263, PHE264, GLN267, ILE977, ILE981, ALA985, ASN1039, LEU1040, ILE1041, and ASN1042. In this computational docking analysis, artemisinin perfectly binds at the active site residues of PfATP6 enzyme (Fig. 5A) which are inconsistent with the results reported earlier^24^,^31^. However, in the mutant PfATP6 proteins (L263D, L263E and L263K) artemisinin binds with different residues when compared to the wild type-artemisinin binding sites (Fig. 5B–D). The difference in the binding residues in mutant protein will indeed change the complementarities of artemisinin bindings with mutant PfATP6 proteins. Shape complementarity and non-covalent interactions are the crucial factors involved in the maintenance of protein-ligand stability. Van der Waals forces, hydrogen bonds, and electrostatic interactions are the primary non-covalent bonds that enhances the protein-ligand affinity. Calculating the non-covalent bond interaction energy is the important parameter in understanding the binding affinity between the protein-ligand molecules. Number of hydrogen bonds arises between the protein-ligand was calculated in Autodock vina. Artemisinin form three hydrogen bonds with wild type PfATP6 protein and one hydrogen bond with L263D, L263E, and L263K mutant protein structures. The binding energies between PfATP6 proteins and the inhibitor molecule artemisinin were calculated to be −8.4 kcal/mol, −7.2 kcal/mol, −7.4 kcal/mol and −7.1 kcal/mol for the wild type, L263D, L263E and L263K complexes respectively (Supplementary Table 1). The wild type-artemisinin complex obtained highest number of hydrogen bonds and binding energy, where as mutant complexes obtained less number of hydrogen bonds and binding energy. From this docking analysis it is concluded that these mutations L263D, L263E and L263K affected the binding affinity of artemisinin with PfATP6 protein and gives a theoretical assessment of the binding mechanism of PfATP6 wild and mutant proteins with antimalarial drug artemisinin.

### PfATP6 mutant proteins structure-based virtual screening and docking studies

**Figure 6:**
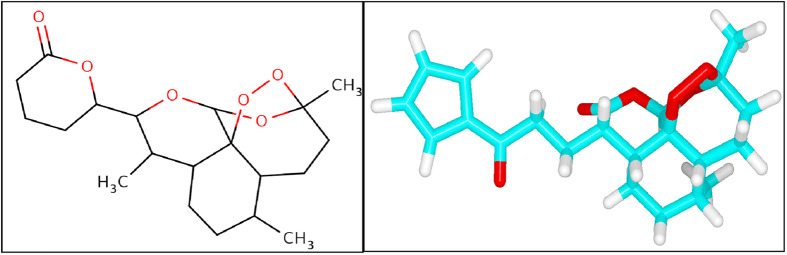
2D and 3D image of compound CID 10595058.

**Figure 7:**
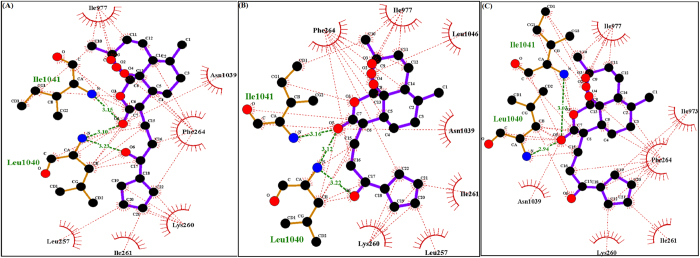
Ligplot analyses of PfATP6 mutant proteins with virtual compounds, green lines indicate the hydrogen bonds and red dotted lines indicates the hydrophobic interactions. (**A**) Ligplot showing interaction between mutant modelL263D and CID 10595058. (**B**) Ligplot showing interaction between mutant modelL263E and CID 10595058. (**C**) Ligplot showing interaction between mutant modelL263K and CID 10625452.

**Figure 8:**
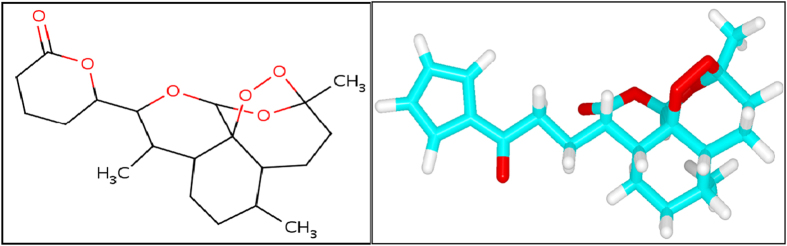
2D and 3D image of compound CID 10625452.

Different studies reported that single point mutations on PfATP6 of the malarial parasite were able to modulate the affinity of artemisinin for the protein and influencing efficacy and toxicity by affecting the artemisinin binding pocket^7^,^20^. In this study also molecular docking analysis resulted that the PfATP6 mutations (L263D, L263E and L263K) affected the artemisinin binding affinity. The resistance of this malarial protein to the traditional artemisinin treatments has led to extensive work in discovering new drugs to treat malaria. *In silico,* virtual screening of chemical compounds is the fastest and most suitable method for identifying novel drug on the basis of target structures^36^,^37^. Virtual screening of the existing compound have advantages over the *de novo* drug design approach because screened lead compounds can be immediately subjected to biological testing. Molecular docking is a robust method primary aim is to calculate binding affinities between a target protein and a ligand. Docking utilizes a defined search pattern to identify the most appropriate orientation of molecules and the binding score that describe the affinity of different conformations^38^,^39^. For virtual screening, we retrieved 393 similar compounds like artemisinin from the PubChem database. Subsequently, docking analyses were performed between mutant PfATP6 proteins (L263D, L263E and L263K) and the screened compounds (Supplementary Table 2). Among the 393 compounds docked with the mutant structures, CID 10595058 binds with L263D and L263E and exhibits high binding affinity of −8.2 kcal/mol and −8.1 kcal/mol respectively (Fig. 6). This compound form three hydrogen bonds with L263D and L263E mutant protein structures (Fig. 7A,B). CID 10625452 (Fig. 8) binds with L263K mutant protein and exhibits high binding affinity −7.9 kcal/mol and forms two hydrogen bonds (Fig. 7C).

### Analyzing molecular dynamics simulation studies

To verify the molecular docking binding affinity results robust or fortune molecular dynamics simulations analysis was performed for PfATP6-Artemisinin, L263D-Artemisinin, L263E- Artemisinin, L263K-Artemisinin, L263D-10595058, L263E-10595058, and L263K-10625452 protein-ligand complexes. The backbone RMSD informations for the wild and mutant type PfATP6-Artemisinin (Supplementary Fig. 1) and PfATP6-Virtually screened lead (Supplementary Fig. 2B) complexes were obtained from the respective trajectories. RMSD of second molecular dynamics run showed more stable convergence in comparison with other two runs was considered for further analysis. The backbone RMSD of wild type and mutant PfATP6-Artemisinin complexes attained stable equilibration after ~10 ns. But high flucations was observed for mutant PfATP6-Artemisinin complexes through out the simulation period and this reveals the existance of bad complementarity between mutant PfATP6 and artemisinin. For PfATP6-Virtually screened lead complexes immediate elevation in the RMSD was observed in the first ~2000 ps due to the kinetic shock introduced during initial dynamic process. Later, the system maintained stable deviation pattern for the remaining simulation period. The overall energy was fluctuated around mean energy with the system equilibrating at one atmospheric pressure and at temperature of 310 K. The molecular dynamics simulation RMSD of the molecules attained stable after ~20000 ps at the equilibrium. The PfATP6-Virtually screened lead complex trajectories attained stable RMSD values around ~20000 ps indicating the stable binding affinity of the protein-ligand complexes. The initial and final structure of the protein-ligand complexes retrived from the trajectories were shown in the Supplementary Figs 3 and 4.

**Figure 9:**
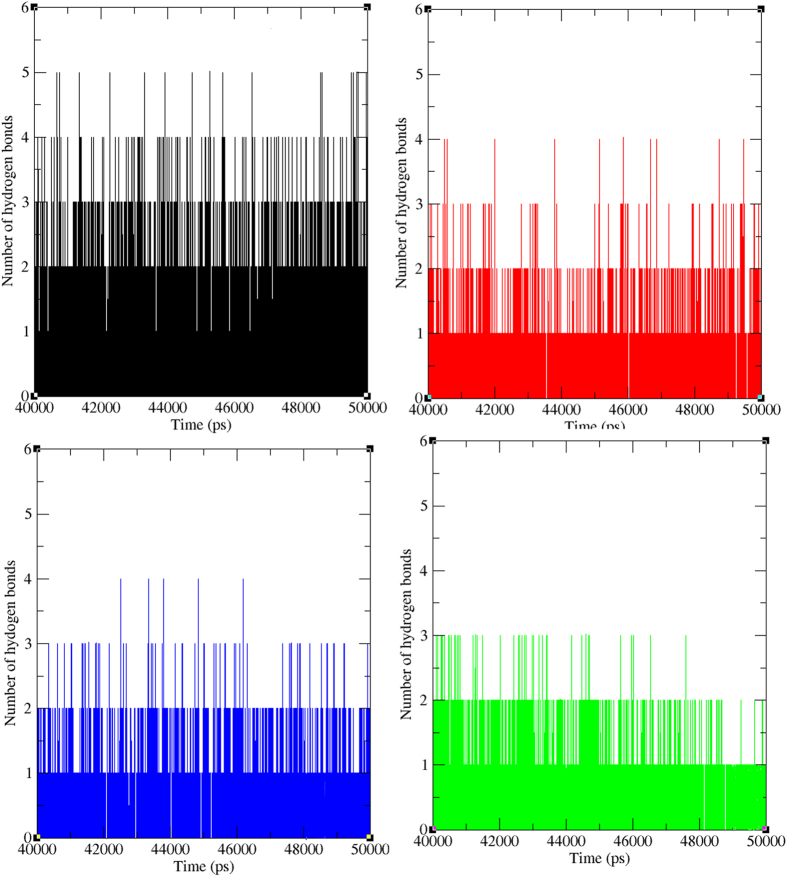
Total number of hydrogen bonds formed between protein-ligand in wild and mutant state. Black, Red, Green and Blue lines indicate the hydrogen bonds formed betweenwild type-artemisinin, L263D-10595058, L263E-10595058 and L263K-10625452 respectively.

Furthermore, to gain the knowledge on the contribution of micro-factors involvement in maintaining the binding affinity between protein-ligand complex, hydrogen bonds between molecules were analysed for wild type and mutant type PfATP6-Artemisinin and PfATP6-Virtually screened lead complexes in the simulation period of last 10 ns. The number of hydrogen bonds formed between wild type and mutant type PfATP6-Artemisinin (Supplementary Fig. 5) and PfATP6-Virtually screened compounds (Fig. 9) were analysed. Wild type PfATP6-Artemisinin obtained one to five hydrogen bonds in the last 10 ns simulation period. But the mutant type PfATP6 L263D, L263E and L263K obtained less number of hydrogen bonds 0 to 2, 0 to 2 and 0 to 3 respectively with anti-malarial drug artemisinin. This shows binding affinity of artemisinin drastically affected in the presence of L263 mutations at the binding pocket of target PfATP6 protein. The numbers of hydrogen bonds formed between L263D-10595058, L263E-10595058 and L263K-10625452 complexes were observed as 1 to 4, 1 to 4 and 1 to 3 respectively. The number of hydrogen bonds between the mutant proteins L263D, L263E and L263K with their respective virtually screened lead compounds is almost similar to the wild type PfATP6-Artemisinin complex. These results provided the evidence that the selected compounds have great calibre to function as strong inhibitors to the specific PfATP6 mutant protein.

**Figure 10:**
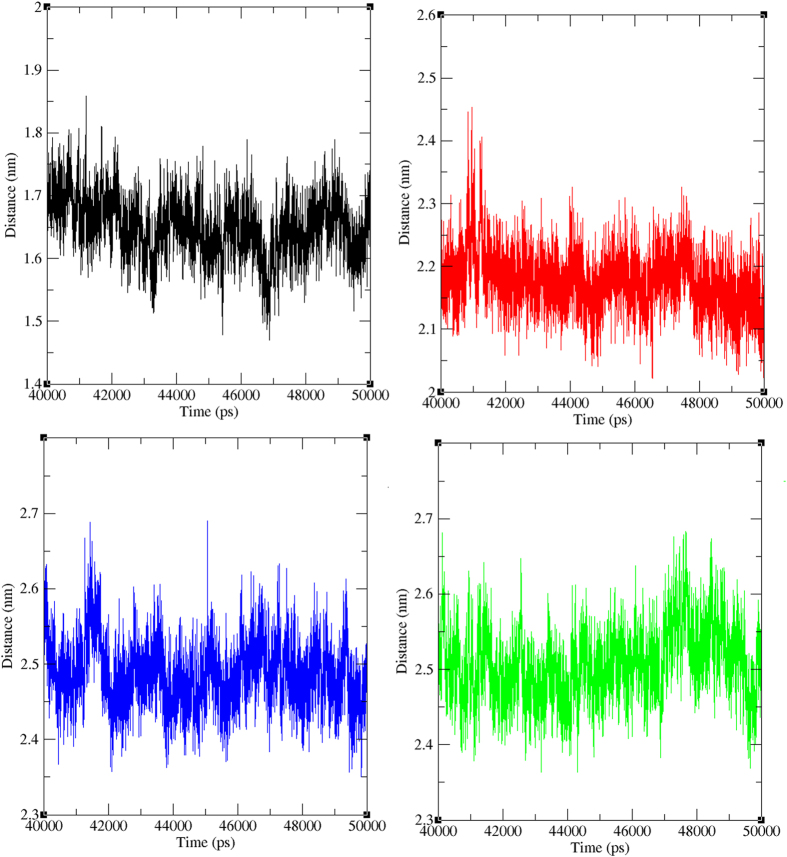
Minimum distance between protein-ligand in wild and mutant state. Black, Red, Green and Blue lines indicate the minimum distance between wild type-artemisinin, L263D-10595058, L263E-10595058 and L263K-10625452 respectively.

The minimum distance between wild type and mutant type PfATP6-Artemisinin (Supplementary Fig. 6) and PfATP6-Virtually screened lead (Fig. 10) complexes were analyzed. The minimum distance between wild type PfATP6-Artemisinin is observed as ~1.5 to ~1.7 nm in the last 10 ns simulation period. But the minimum distance of mutants L263D, L263E and L263K with artemisinin were observed high as ~2.5 to ~2.7 nm, ~2.4 to ~2.6 nm, and ~2.4 to ~2.7 nm respectively. The minimum distance between L263D-10595058, L263E-10595058 and L263K-10625452 mutant proteins with virtually screened lead complexes is observed as ~2.1 to ~2.3 nm, ~2.4 to ~2.6 nm, and ~2.4 to ~2.6 nm respectively. The minimum distance between the mutant proteins and their respective lead compounds is much higher than the wild type PfATP6-Artemisinin complex. Even though the distances are slightly high but mutant PfATP6 proteins and virtually screened compounds are maintained stable contact and distance throughout the simulation period.

**Figure 11:**
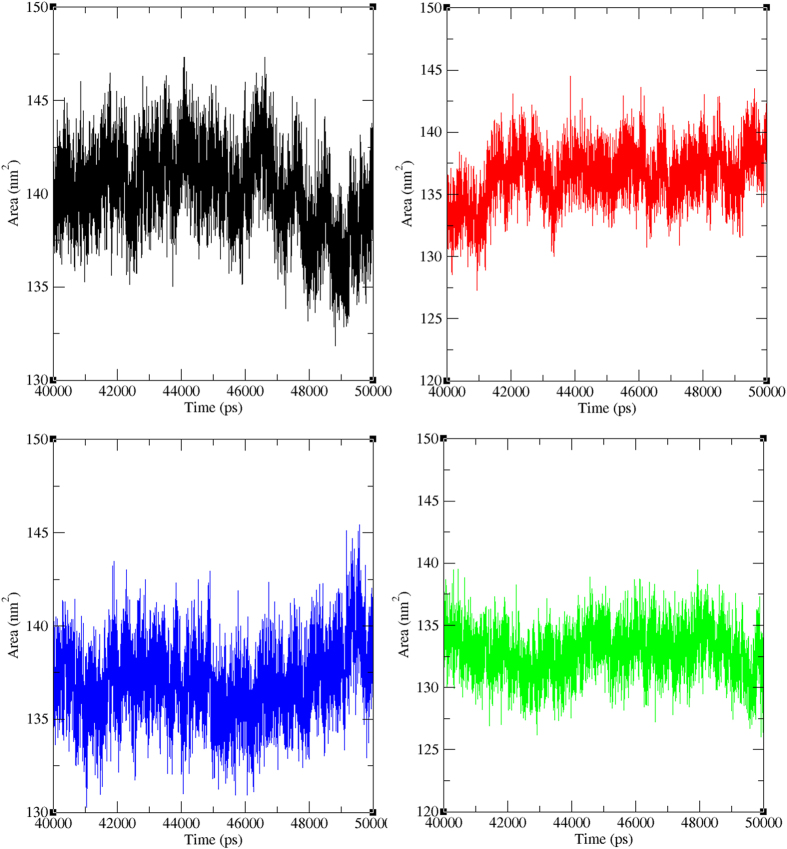
Solvent accessible surface area (SASA) analysis of wild and mutant PfATP6 proteins. Black, Red, Green and Blue lines indicate wild type, L263D, L263E, and L263K PfATP6 proteins respectively.

The area measured by the probe rolling on the surface of the proteinis referred as the solvent-accessible surface area (SASA). This solvent effect is important in maintaining protein stability and act as a driving force for protein folding is to be found in the burial of hydrophobic region. Likewise, this charged effect accompanies protein-ligand interaction processes and reason for protein orientation maintenance. In molecular dynamic simulations, this accessible surface area of molecules are measured by using a sphere of water molecules^40^. SASA was calculated for both the wild type and mutant PfATP6 proteins. From Fig. 11, it was observed that the wild-type PfATP6 have SASA of ~133 nm^2^ to ~147 nm^2^ in the last 10 ns simulation period but the mutant complexes L263D, L263E and L263K obtained less SASA of ~130 nm^2^ to ~142 nm^2^, ~132 nm^2^ to ~145 nm^2^, and ~127 nm^2^ to ~138 nm^2^ respectively. Compared with the wild type, all three mutated proteins obtained less SASA. This indicates that there might be orientational change in the protein surface because of the amino acid residue shift from the accessible area to buried region.

## Discussion

Owing to the emerging resistance to existing antimalarial drugs there is a need for discovering novel drugs to treat malaria. Medicines for malaria venture and other product development partnerships initiated various drug discovery programs that reach milestone development of potential new antimalarial lead molecules^41^,^42^, which are currently under clinical trials. Despite these successes, it is important to maintain early phase drug discovery to prevent the antimalarial drug development pipeline from draining^43^,^44^. Rational post genomic drug discovery is based on the protein structure based screening of compounds from large chemical libraries either by high throughput or virtual screening methods. The primary focus of protein structure based drug design approach is the identification of a suitable drug. The target structure must essential for the survival of the parasite and sufficiently different from the similar family of proteins in the host species to be inhibited specifically/selectively. The emergence of resistance first-line antimalarial treatment drug artemisinin has recently begun to affect the processing of patients suffering from *P. falciparum* infections in western Cambodia^8^,^9^. If the artemisinin resistance spreads, large population will be affected in public health, as there is no suitable drug to treat *P. falciparum*. Therefore, there is an immediate requirement in understanding the mechanisms of action of artemisinin because this information may contribute to set a pipeline for monitoring emerging resistance and further for the development of new antimalarial drugs^45^.

Protein 3D modeling and molecular docking simulations studies of the PfATP6 sequence suggested that artemisinin might interact directly with malarial PfATP6^21^,^29^,^30^. So far, few genes have been proposed to be associated with reduced sensitivities to artemisinin, but till now none of the hypothesis has been proved^46^. Even though several studies have been conducted to elucidate the mechanism of artemisinin binding but the predicted mechanism of action is not completely understood, since PfATP6 molecular biology is the lack of phylogenetically close 3D structures that could serve as template.

In this study, we followed a systematic computational approach to explore the mechanism of binding action of artemisinin with PfATP6 protein and also elucidated the molecular basis of the artemisinin resistance to L263 mutations. Since the target PfATP6 protein structure yet not resolved, PfATP6 3D structure constructed through homology modelling by using 3TLM as a template structure. But in previous studies 1IWO and 2DQS protein’s 3D structure were used as a template for the 3D structural construction for malarial PfATP6 protein. Sequence similarity and phylogenetic comparison between malarial PfATP6, 3TLM, 1IWO and 2DQS protein sequences revealed that malaria PfATP6 and 3TLM protein sequence are evolutionarily closest. Our ultimate goal of this study is to elucidate the mechanistic action of artemisinin binding with wild and L263 mutant PfATP6 enzyme in open conformation model. Hence in this study 3TLM structure was used as the template to construct malarial PfATP6 3D structure. For structural analysis, we modelled the mutant (L263D, L263E and L263K) structures by using spdbv software. Following that, energy minimization was performed for the wild type and mutant PfATP6 proteins using steepest descent force field to maintain the geometry of the modelled protein. Next, docking analyses were performed between the wild type and mutant PfATP6 protein with drug artemisinin. Important factors maintaining the protein-ligand affinity were analysed. Binding energy between the mutant protein and drug revealed less binding affinity for the mutant structures with antimalarial drug artemisinin. Notably, artemisinin interacting residues differ in the mutant proteins and formed less number of hydrogen bonds than the wild type PfATP6 protein. These results confirmed that structural changes occurred in the PfATP6 protein because of L263 mutations. To identify a suitable inhibitor, structure-based virtual screening and docking analysis was performed. A total of 393 compounds that are structurally similar to antimalarial drug artemisinin were retrieved from the PubChem database. Each compound was individually docked with L263D, L263E and L263K mutant structures of malarial PfATP6 protein. CID 10595058 binds with L263D and L263E and this compound forms three hydrogen bonds with each of the mutant structure. CID 10625452 binds with L263K and forms two hydrogen bonds with L263K mutant structure.

Furthermore, we performed 50 ns MD simulation analysis on the wild type and mutant type PfATP6-Artemisinin and PfATP6-Virtually screened lead complexes. MD simulation analysis provides insights into the protein-ligand binding affinity and interactions at the atomic scale. Basic parameters RMSD, hydrogen bond formation, minimum distance and SASA were examined on last 10 ns of protein-ligand simulation trajectories for PfATP6-Artemisinin, L263D-Artemisinin, L263E-Artemisinin, L263K-Artemisinin, L263D-10595058, L263E-10595058 and L263K-10625452 complexes. In the molecular stability change analysis all the mutant PfATP6-Artemisinin and mutant PfATP6-Virtually screened lead complexes obtained higher RMSD values than the wild type PfATP6-Artemisinin complex. Hydrogen bond formation between the protein and ligand served as the main contributor in maintaining the affinity and stability of the molecules. The substitution of amino acid changed the electrostatic potential in PfATP6 protein surface and affected the artemisinin binding. However, the virtually selected lead compound obtained strong binding affinity towards the mutant proteins and having the capability to maintain a stable number of hydrogen bonds during the simulation period. In all three mutant complexes (L263D-10595058, L263E-10595058, and L263K-10625452) almost same number of hydrogen bonds was observed as similar to wild type-artemisinin complex. The minimum distance formed between mutated proteins and the virtually screened potential lead compounds were slightly higher than the wild type PfATP6-artemisisn complex. Eventhough the distance is higher but it would not affect the affinity between molecules since the observed distance falls under the cut-off radii of non-bonded bonds formation limits. In SASA analysis, less solvent accessible surface were observed in all three mutant proteins in compare with wild type PfATP6 protein. Less accessible areas in mutant proteins might affect the probability of interactions between PfATP6 and artemisinin. Subsequently SASA results revealed that the occurance of mutations in the PfATP6 protein changed the hydrophilic and hydrophobic areas of the mutant PfATP6 proteins and affected the artemisinin binding.

## Conclusion

Despite increased support for malaria control over the past decade, the malaria burden remains high in many endemic countries. Prompt treatment with artemisinin based combination therapy targeted towards those confirmed to have malaria is a key malarial control strategy. Although artemisinin is a primary and quickly responding antimalarial drug, the curative rate of patients treated with *P. falciparum* malaria is decreased because of the emerging drug resistance. Due to the high propensity of the parasite to become drug resistant the need for new antimalarial drugs will persist until the malarial parasites are eventually eradicated. Because of several factors, the three-dimensional structure of plasmodial proteins is not yet resolved through the physical approach. However, recent advancement in the computational techniques will helpful partially to overcome the traditional approach difficulties. This study is such one which describes the three-dimensional structure of malarial PfATP6 protein and then different *in silico* strategies were used to evaluate the resistant mechanism with artemisinin and well as subsequent rational approaches for identifying new lead molecules. The two compounds identified in this study can be tested *in vitro* and *in vivo* experiments as possible candidates for the designing of potential antimalarial medicines.

## Materials and Methods

### Protein homology modeling and refinement

The PfATP6 enzyme sequence of *P. falciparum* was obtained from UniProt^22^ database. The BLAST server^47^ was used to search the closest sequence of PfATP6 in the RCSB Protein Data Bank. The high proportion of amino acid sequence identity between the target PfATP6 and endoplasmic reticulum Ca^2+^-ATPase (Serca) from bovine muscle (45% homology) indicates that crystal structure of the latter are good models, can be used as templates. The SERCA 3D structure of bovine muscle was obtained from the RCSB Protein Data Bank (PDB: 3TLM)^23^. The sequence alignment of PfATP6 with SERCA as a reference was performed using ClustalW 2.0.1 by applying the default parameters. Protein reconstruction was achieved with Modeller 9.15 on the entire PfATP6 sequence^27^,^28^. The refinement was made with the loop model class. The quality of each Modeller refined model was evaluated with PROCHECK (version 3.5.4)^29^. The final model was chosen from both the PROCHECK results and the Modeller energy score. Hydrogen atoms were added, and the PfATP6 structure was minimized using the Gromos96 43a1 force field^48^,^49^.

### Docking and virtual screening

AutoDock is the widely accepted molecular docking programs and requires a set of preparation steps for general screening^50^. Included in this process are preparations of acceptable ligands and a receptor macromolecule, calculation of maps and creation of folders for each ligand. AutoDock Vina is the updated version of AutoDock program with improved molecular docking and virtual screening strategies and was approximately two orders of magnitude faster than AutoDock4^32^. Vina uses a gradient optimisation method in its local optimisation procedure. The calculation of the gradient effectively gives the optimisation algorithm a “sense of direction” from a single evaluation. By utilizing the multi threading, Vina can produce fastest calculationon docking analysis by taking advantage of multiple CPUs or CPU cores. The assessment of the speed and accuracy of Vina during the flexible re-docking of 190 protein-ligand complexes demonstrated that the AutoDock4 training set was processed almost two orders of magnitude faster and with a significant improvement in binding mode prediction accuracy. Furthermore, Vina can further reduce computational time by utilising multiple CPU cores. The tool, VcPpt was used for high throughput virtual screening of compounds, VcPpt is an independently developed software package for flexible protein-ligand docking by the Biochem Lab solution.

### Molecular dynamic simulations protocol

Molecular dynamics simulations of wild-type and mutant protein-ligand complexes were performed using Gromacs 5.0 software^51^. The force field used for energy minimization was Gromos96 43a1^48^,^49^. The structures were solvated using a simple point charge (SPC) water box with a dimension of 52.0 Å. At physiological pH all the protein-ligand complexes obtained negative charges and to neutralize the system counter ions (Na+) were added in to the simulation box. Then, all the atoms in the box were energy minimized by applying the steepest descent algorithm. Next to the energy minimization, furthermore three steps have been followed i.e heating the system, equilibrating the system and finally the production of trajectories. The NPT (constant number of particles, pressure, and temperature) ensemble and then the NVT (constant number of particles, volume and temperature) ensemble was performed at 300 K for 50000 ps^52^. Finally, the production of MD simulation was performed for 50 ns at 300 K. The covalent bonds were constrained by the Linear Constraint Solver (LINCS) algorithm^53^. The Particle Mesh Ewald (PME) method was used to calculate electrostatic interactions^54^. The cut-off radii for Coulomb interactions and van der Waals were fixed at 10.0 Å and 14.0, respectively.

By utilizing GROMACS utilities the trajectories obtained from each simulations were completely analyzed^55^. The utilities g_rms, g_hbond, g_mindist and g_sas were used to plot RMSD, the number of hydrogen bonds formed between molecules, the minimum distances between molecules and solvent-accessible surface area (SASA) of proteins respectively. The numbers of hydrogen bonds and the minimum distance formed between protein-ligand complexes were calculated to explain the stability of the complexes. SASA analysis was performed to identify the traceable area of a molecule and all graphs were generated using the XM grace tool^56^.

## Additional Information

**How to cite this article**: N., N. *et al*. Mechanism of artemisinin resistance for malaria PfATP6 L263 mutations and discovering potential antimalarials: An integrated computational approach. *Sci. Rep.* **6**, 30106; doi: 10.1038/srep30106 (2016).

## Supplementary Material

Supplementary Information
