# Supplementary material for: Mechanism of artemisinin resistance for malaria PfATP6 L263 mutations and discovering potential antimalarials: An integrated computational approach
Source: Sci Rep. 2016 Jul 29;6:30106. doi: 10.1038/srep30106 (PMC4965867; doi:10.1038/srep30106)
Supplement: Supplementary Information [file srep30106-s1.pdf]

Mechanism of artemisinin resistance for malaria PfATP6 L263 mutations and discovering potential antimalarials: An integrated computational approach

Nagasundaram. N, George Priya Doss. C, Chiranjib Chakraborty, Karthick V, Thirumal Kumar D, Balaji V, Siva R, Aiping Lu, Ge Zhang, Hailong Zhu*

Supplementary Information

Supplementary Figure 1. Backbone Root Mean Square Deviation (RMSD) of wild type malarial PfAPT6 protein and mutants in complex with drug artemisinin (ART). The ordinate is RMSD (nm) and the abscissa is the time (ps). Black, Red, Green and Blue lines indicate wild type, L263D, L263E, and L263K proteins respectively.

Supplementary Figure 2. Backbone Root Mean Square Deviation (RMSD) of malarial PfAPT6 proteins. The ordinate is RMSD (nm), and the abscissa is the time (ps). Black, Red, Green and Blue lines indicate wild type, L263D, L263E, and L263K protein respectively.

Supplementary Figure 3. Initial (0 ns) and final structures (50 ns) of wild type and mutant type PfATP6-Artemisinin complexes.

Supplementary Figure 4. Initial (0 ps) and final structures (50 ns) of mutant type PfATP6-Virtually screened complexes.

Supplementary Figure 5. Total number of hydrogen bonds formed between PfATP6-artemisinin in wild and mutant state. Black, Red,Green and Blue lines indicate the hydrogen bonds formed between wild type-artemisinin, L263D- artemisinin, L263E-artemisinin and L263K-artemisinin respectively.

Supplementary Figure 6. Minimum distance between protein-ligand in wild and mutant state. Black, Red, Green and Blue lines indicate the minimum distance between wild type-artemisinin, L263D- artemisinin, L263E- artemisinin and L263K- artemisinin respectively.

Supplementary Table 1 Binding energies of PfATP6 wild and mutant proteins with antimalarial drug artemisinin.

Supplementary Table 2 Virtual compounds with highest binding energy with PfATP6 mutant proteins

Supplementary Figure 1

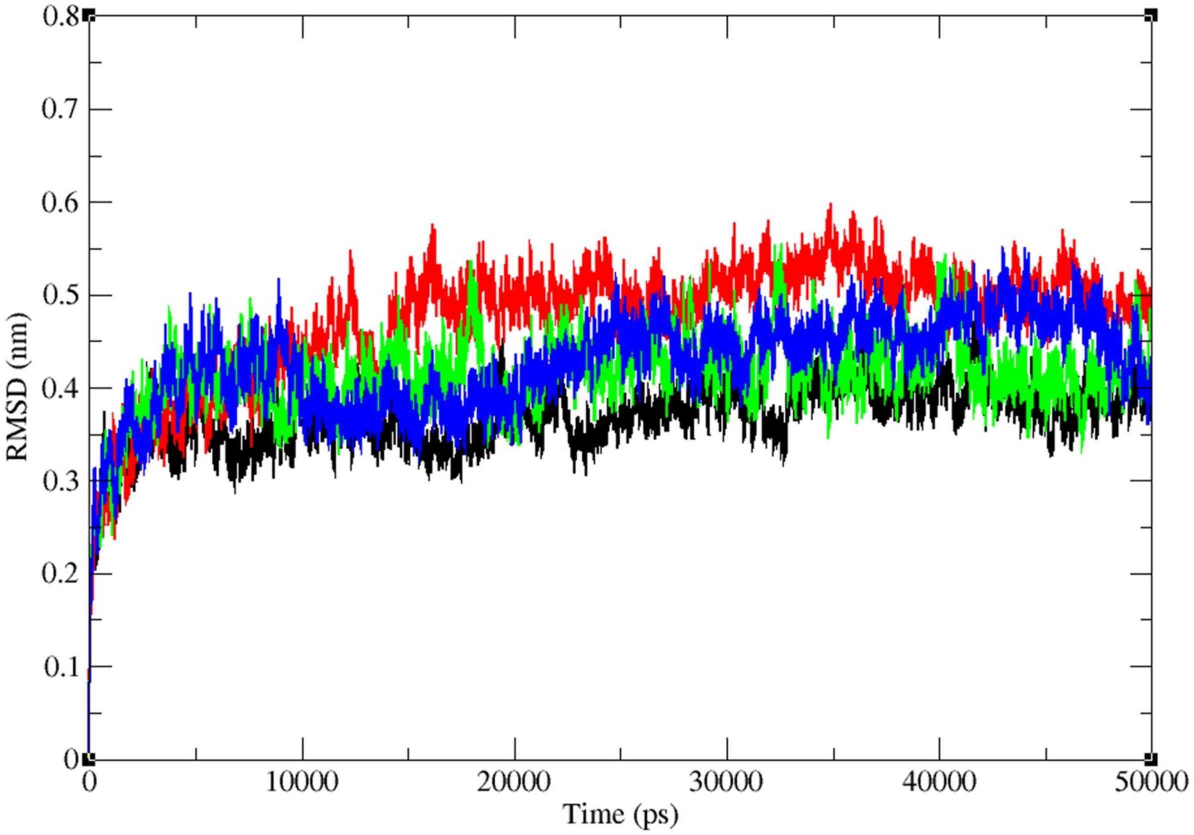

Supplementary Figure 2

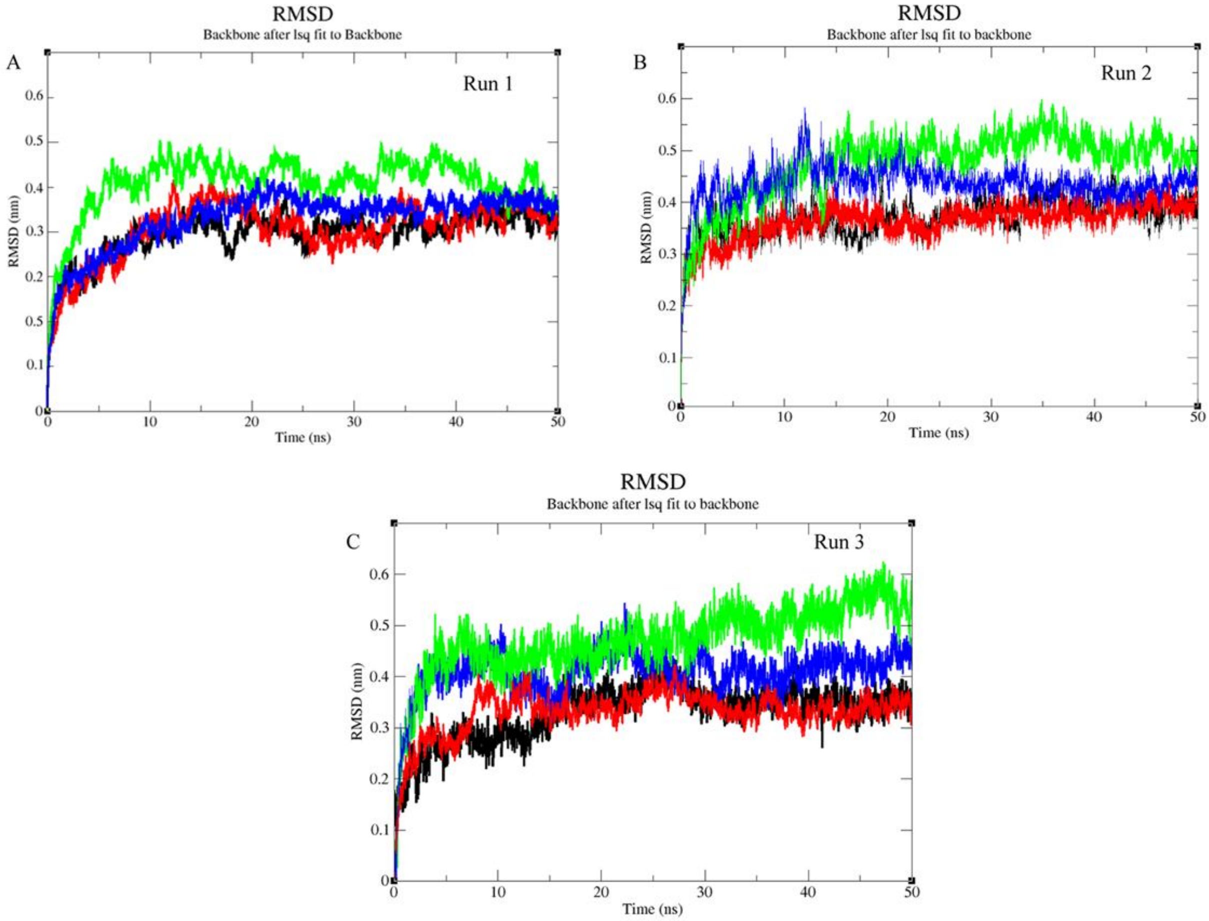

Supplementary Figure 3

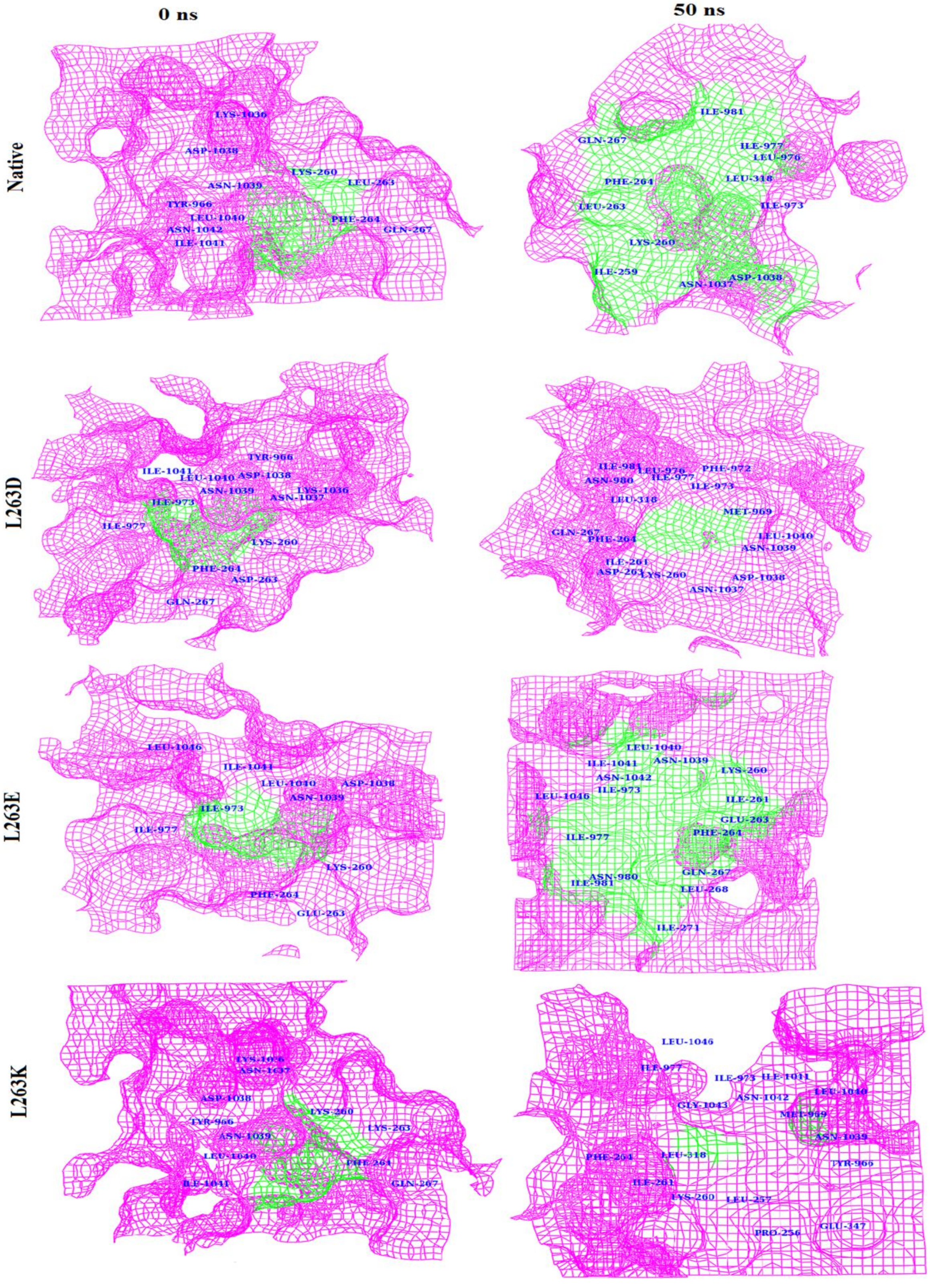

Supplementary Figure 4

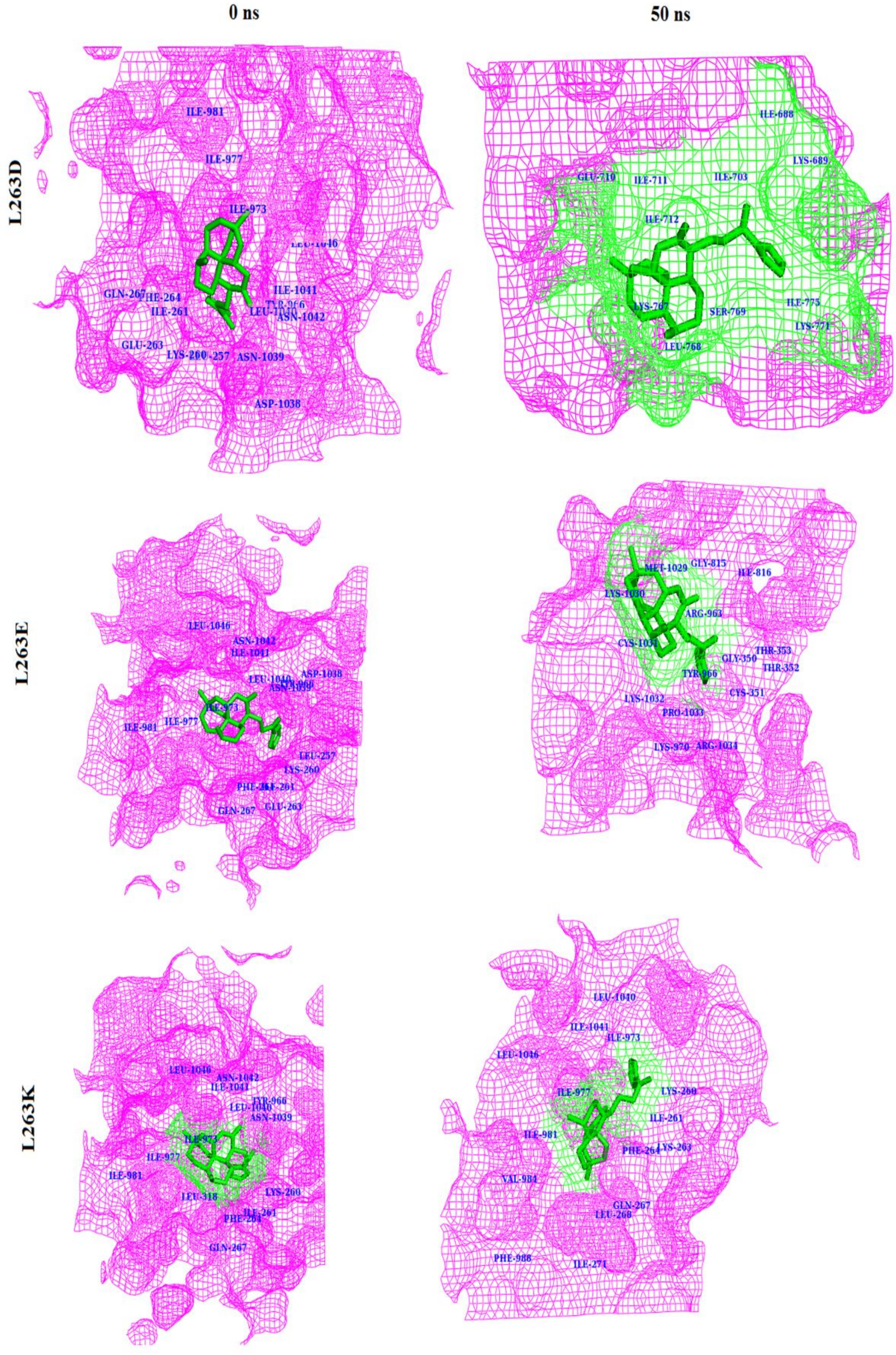

Supplementary Figure 5

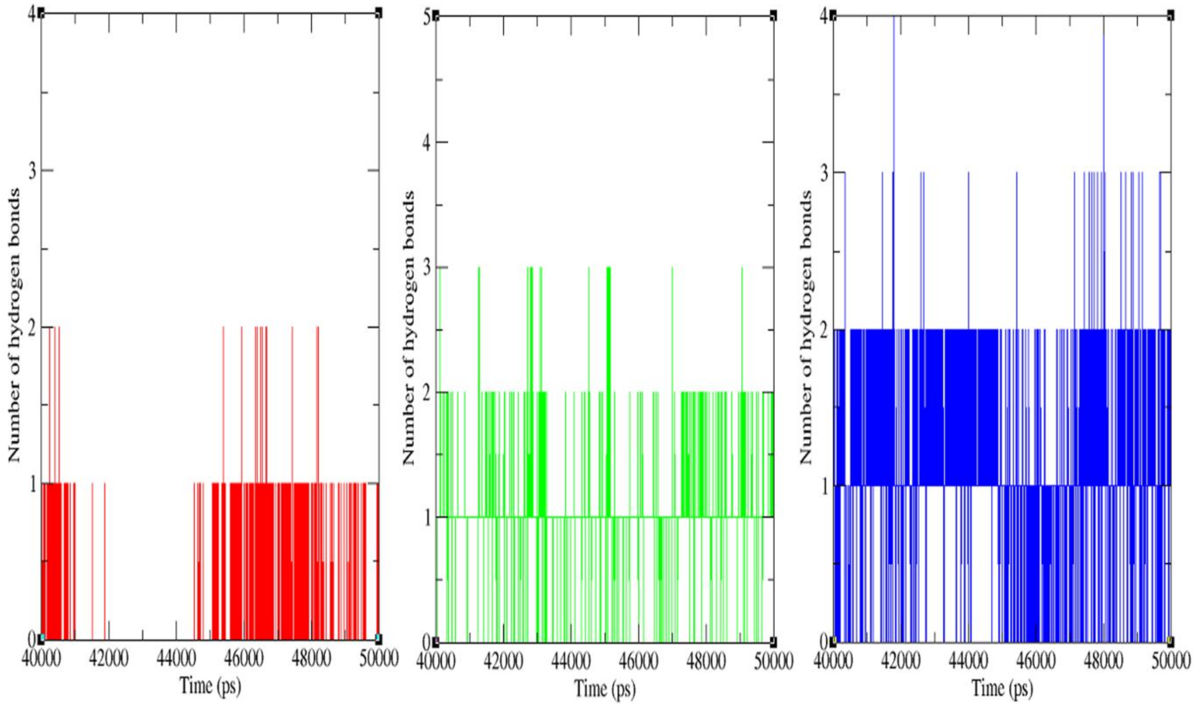

Supplementary Figure 6

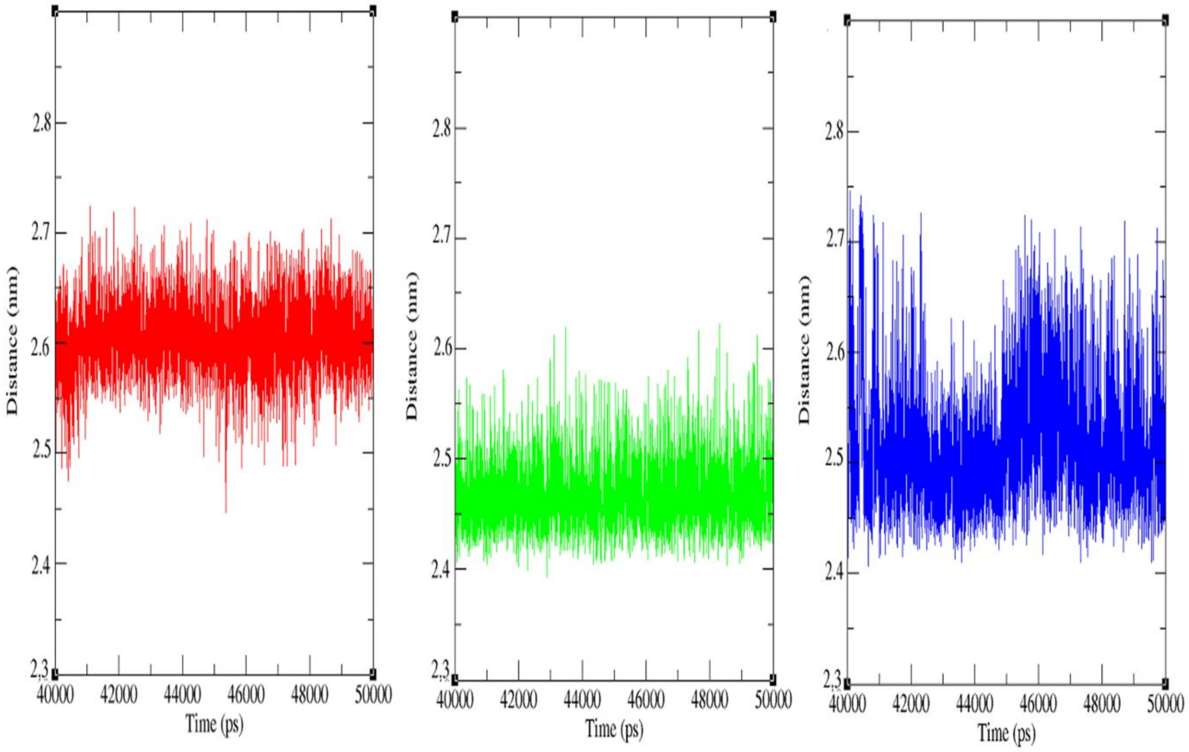

Table S1 Binding energies of PfATP6 wild and mutant proteins with antimalarial drug artemisinin.

**Wild & Mutant proteins**	**Binding Energy(Kcal/mol)**	**Number of H-Bonds**	**Residues forming H-Bonds with Ligand**	**Interacting Residues**
Wild type protein	-8.4	3	Leu1040, Ile1041	LYS260, LEU263, PHE264, GLN267, ASP1038, ASN1039, LEU1040, ILE1041
L263D	-7.2	1	Leu1040	LYS260, PHE264, GLN267, ASP1038, ASN1039, LEU1040, ILE1041
L263E	-7.4	1	Leu1040	LYS260,GLU263, PHE264, ILE973, ASN1039, LEU1040, ILE1041
L263K	-7.1	1	Leu1040	LYS260, PHE264, GLN267, ASP1038, ASN1039, LEU1040, ILE1041

Table S2 Virtual compounds with highest binding energy with PfATP6 mutant proteins

PfATP6 Mutant Proteins	Binding Drug CID	Binding Energy(Kcal/mol)	Number of H-Bonds	Residues Forming H-Bonds with Ligand	Interacting Residues
L263D	10595058	-8.2	3	LEU1040, ILE1041	LEU257, LYS260, ILE261, PHE264, , ILE977, ASN1039, LEU1040, ILE1041
L263E	10595058	-8.1	3	LEU1040, ILE1041	LEU257,LYS260, ILE261, PHE264, , ILE977, ASN1039, LEU1040, ILE104, LEU1046
L263K	10625452	-7.9	2	LEU1040, ILE1041	LYS260, ILE261, PHE264, ILE973, ILE977, ASN1039, LEU1040, ILE104,
